# Rural-Urban Differences in Social Connectedness Among Adult Foster Home Residents in Oregon

**DOI:** 10.1093/geroni/igaa057.085

**Published:** 2020-12-16

**Authors:** Ozcan Tunalilar

**Affiliations:** Portland State University, Portland, Oregon, United States

## Abstract

Social isolation has been linked to negative health outcomes, especially among older adults. Although ability to maintain social contact and existing ties to one’s community is a primary benefit of receiving long-term supports and services in a community-based setting, few studies explored how geography might shape these residents’ access to family members and friends. The current study explores this question in the context of adult foster homes (AFH), a type of family-style residential care licensed for five or fewer unrelated adults. Using cross-sectional data collected annually from 1,500 AFHs between 2015 and 2020, the study examines whether older adults residing in rural and urban AFHs in Oregon differ in terms of levels of distinct types of contact with their existing social networks. AFHs were designated as rural/urban at the zip code level using the definitions provided by the state coordinating organization for rural health. Results from negative binomial regression models show that rural residents were significantly less likely to receive help from their family members and friends in getting to medical appointments or outside activities (e.g., meals, walks, shopping) or receive social visits or phone calls compared to their urban counterparts. Rural and urban residents had similar levels of help with personal care and taking medications. These results remained unchanged after accounting for a set of home (e.g., Medicaid contract) and resident characteristics (e.g., acuity). These findings suggest important public health implications for improving rural residents’ social connectedness and interventions aiming at improving social participation in long-term care residents.

